# Preparation of Anionic Surfactant-Based One-Dimensional Nanostructured Polyaniline Fibers for Hydrogen Storage Applications

**DOI:** 10.3390/polym15071658

**Published:** 2023-03-27

**Authors:** Hatem A. Al-Aoh, Nacer Badi, Aashis S. Roy, Abdulrhman M. Alsharari, Salah Abd El Wanees, Abdulrahman Albaqami, Alex Ignatiev

**Affiliations:** 1 Department of Chemistry, Faculty of Science, University of Tabuk, Tabuk 71491, Saudi Arabia; 2 Department of Physics, Faculty of Science, University of Tabuk, Tabuk 71491, Saudi Arabia; 3 Renewable Energy & Energy Efficiency Center, University of Tabuk, Tabuk 71491, Saudi Arabia; 4 Department of Chemistry, S. S. Tegnoor Degree College, Kalaburagi 585105, India; 5 University College of Umluj, University of Tabuk, Tabuk 48266, Saudi Arabia; 6 Department of Physics, University of Houston, Houston, TX 77204, USA

**Keywords:** polyaniline fiber, hydrogen storage, surfactant, nucleation, conductivity

## Abstract

Polyaniline fibers were prepared in the presence of anionic surfactant in an ice medium to nucleate in one dimension and were compared to bulk polyaniline prepared at an optimum temperature. Fourier-transform infrared spectroscopy (FTIR) and X-ray powder diffraction (XRD) were used to investigate the structural analysis of the prepared samples. A conductivity study reveals that polyaniline fibers have high conductivity compared to bulk polyaniline. Hydrogen storage measurements confirm that the polyaniline fibers adsorbed approximately 86% of the total actual capacity of 8–8.5 wt% in less than 9 min, and desorption occurs at a lower temperature, releasing approximately 1.5 wt% of the hydrogen gases when the pressure is reduced further to 1 bar.

## 1. Introduction

The use of hydrogen instead of gasoline as an automobile fuel has the potential to lower global greenhouse gas emissions. In comparison to other fossil fuels or hydrocarbon fuels, it has an energy density per kilogram of 142 MJ [1]. Hydrogen can be used in a fuel cell with oxygen to create electricity and water as byproducts, or it can be burned in an internal combustion engine [2]. A hydrogen fuel cell vehicle (FCV) can travel 400 km on 4 kg of hydrogen, which is equivalent to the distance any conventional gasoline-powered vehicle can travel on 24 kg of petroleum. In order to achieve this, hydrogen must be charged into a car and stored prior to use. However, the use of hydrogen fuel in transportation applications presents significant challenges. Therefore, it is crucial to create appropriate noble materials for effectively and safely storing hydrogen [3]. A number of variables, including the conducting polymer’s specific surface area, structural morphology, acidity, environmental stability, type of gases, pressure, temperature, etc., affect its ability to adsorb substances [4,5].

Conducting polymers are among the first materials to successfully demonstrate hydrogen electrosorption (HES). The HES could be reversed and undesirable lattice expansion was possible with the hydride interstitial type of NHx -H+ bonding [6,7,8]. Specific surface area, morphology, porous structure, temperature, and pressure are a few of the variables that affect how readily hydrogen is absorbed by conducting polymers. Due to its straightforward synthesis, low price, and excellent environmental stability, polyaniline has recently grown in popularity among conducting polymers [9,10,11].

Additionally, the storage medium must be reversible and maintain its storage effectiveness even after receiving multiple hydrogen charges. Therefore, it is crucial to create materials that can hold and release gas at standard pressure and temperature. For the majority of the last ten years, the preferred material for hydrogen storage has been metal hydrides and metal hydride complexes. While liquid hydrogen only has a density of 4.2 H atoms per cm^3^, metal hydrides, specifically magnesium hydrides, have the capacity to store 6.5 H atoms per cm^3^ [12]. This benefit offers the possibility of achieving high hydrogen gravimetric uptake while still maintaining a safe vessel. Due to their low atomic weight, metal hydride complexes of lithium, aluminum, beryllium, magnesium, and sodium make up the majority of this class of materials. Atoms of hydrogen are chemically bonded within the interstitial sites of the lattice complex after dissociating on the metal surface and being absorbed into the material [12]. The activation energy necessary to rupture the interstitial bonds and release the hydrogen atoms from the substance is the “bottleneck” with this material.

In an effort to overcome the limitations of metal hydrides, organic polymers have undergone extensive research for hydrogen storage. The use of polyaniline (PANI) for hydrogen storage has attracted interest due to its special electronic properties as a conducting polymer [13]. Sunghun Cho et al. reported a recent development in conducting polymers for hydrogen storage and fuel cell applications in 2020 [14]. The most frequent substance deposited into a polymer substrate is metal nanoparticles. Zhiyang Liu et al. reported hydrogen storage via metal atom functionalization in 2021 [15]. Simply because hydrogen is the only physisorption process used by polymers, they are able to overcome the high desorption temperature and kinetics issue. There are no chemical bonds created between the adsorbent and the hydrogen molecule because only physical adsorption is taking place. It is simple to break the weak Van der Waals forces involved in adsorption and desorption, releasing hydrogen while doing so very quickly [16]. The challenge with these materials is to design pores with enough space for hydrogen to adsorb under ambient conditions. The pore size needs to be smaller than 10^−6^ m in diameter between the pore walls to be considered microporous [8]. Another important consideration is the material’s specific surface area, which rises as pore size falls [16]. Low temperatures (77 K) are needed to adsorb a sufficient amount of hydrogen due to the low adsorption enthalpy of organic polymers, which also happens to be the temperature of liquid nitrogen [16]. Because of the improved accuracy at this temperature, it is also possible to test a material’s precise surface area at liquid nitrogen temperature. When determining the surface area of nitrogen adsorption curves, the Brunauer–Emmett–Teller (BET) equation is frequently used [17].

Due to the inconsistent reporting of hydrogen storage and the lack of reproducibility among publications, the hydrogen storage capacities of various materials are a very challenging topic to discuss. Previous studies claimed that the gravimetric storage capacities of polyaniline and magnesium hydride derivatives ranged from 10 to 20 weight%. However, those studies have since been shown to be false and invalid; errors stem from the sensitivity of the experimental setup and incorrect reporting of the units for hydrogen storage capacities. Storage capacities are also reported at various temperatures, such as liquid nitrogen’s 77 K temperature, and if one is not careful, they could be deceiving.

Modern storage technologies, even those that are completely new, frequently fall short of meeting all the requirements for hydrogen storage, including high storage density, low desorption energy requirements, safety, purity, dynamics, low material costs, etc. There does not seem to be a single solution that can meet all the requirements. A substance known as polyaniline (PANI) has demonstrated the ability to adsorb and desorb hydrogen, particularly when in the form of a nanostructure. The main opportunity is the potential for significant weight-per-percentage storage of hydrogen at room temperature or slightly above it, under moderate pressure, and another opportunity is the low cost of bulk polyaniline material. Researchers have measured the reversible hydrogen gas storage in doped (metallic) forms of organic conducting polymers (also known as “synthetic metals”), such as polyaniline and polypyrrole, and have reported values ranging from 2.5 to 6 wt% of hydrogen adsorption for various nano-preparations of polyaniline [17,18]. However, the results have come under fire for not using adequate measurement techniques.

The primary goal of the research is to store large amounts of hydrogen at optimal temperature and pressure [19]. There have been several reports on the hydrogen storage properties of polyaniline (PANI) and polypyrrole (PPy) due to the formation of nanostructures and large physio-chemisorption sites [20,21,22]. PANI is an intrinsically conducting polymer whose conductivity can be altered by the addition of organic or inorganic acids [23,24,25,26]. Under certain conditions, acid treatment modifies the physiochemical properties of polyaniline, causing the formation of a pattern of a specific nanostructure with small pores (<30 nm), and these properties are more desirable for hydrogen storage [27]. Therefore, we attempted to investigate polyaniline with various morphologies for hydrogen storage applications. Polyaniline fibers were prepared at ice temperatures and characterized using Fourier-transform infrared spectroscopy (FTIR), X-ray powder diffraction (XRD) for structural analysis and surface morphology by scanning electron microscopy (SEM), contact angle, and hydrogen sorption measurements with Hy-PCTPro Energy’s 2000 sorption equipment. The DC conductivity study was carried out in 10mm plate form using a two-probe method, and the dielectric study was carried out using a Hoiki impedance analyzer.

## 2. Materials and Methods

All reagents used for the synthesis of polyaniline are of analytical grade. The chemicals used for the synthesis of polyaniline fibers are aniline monomer having a molecular weight of 93.13 and purity of 99%, hydrochloric acid (HCl) (Molecular Weight: 36.46), ammonium persulphate ((NH_4_)_2_S_2_O_8_) (Molecular Weight: 228.20 and purity 98%), hydrogen peroxide solution (H_2_O_2_) with 35% concentrated and mol. wt 34.01, 36.46% of concentrated hydrochloric acid, toluene-4-sulfonic acid monohydrate (C_7_H_8_O_3_S·xH_2_O) with mol. wt 172.20 and 98.6% purity, ammonium persulphate with 99.9% purity, and acetone with 99.8% purity were procured and used as it is from Sigma-Aldrich Pvt Lt, Bengaluru, India.

### 2.1. Synthesis of Polyaniline

The oxidation polymerization method, as reported previously, was used to make polyaniline. By continuously stirring 100 mL of 0.1 M aniline and 100 mL of 1 M HCl prepared in deionized water, an aniline hydrochloric acid solution was produced. After that, 100 mL of 0.25 M ammonium persulphate (APS) is added gradually while being stirred continuously. The reaction mixture was kept at 5 °C to react steadily for 24 h without further stirring after the APS has been fully incorporated, producing a green precipitate. The precipitate was cleaned with HCl and acetone, then filtered before being allowed to dry at 50 °C to achieve a constant weight [28,29].

### 2.2. Synthesis of Polyanilinefibers at Ice Temperature

A 100 mL of the 0.1 M aniline aqueous solution was mixed with 100 mL of 1 M HCl, and 100 mL of 1 M toluene-4-sulfonic acid monohydrate (PTSA) solution prepared in deionized water with constant stirring to form an aniline hydrochloro- toluene-4-sulfonic acid solution and transferred to an ice bath allowing the solution mixture to penetrate the ice pores. Subsequently, 100 mL of 0.25 M ammonium persulphate (APS) is added slowly at 0 °C temperature with continuous stirring. Once the APS has been completely added to the reaction mixture, it is kept in the freezer to react steadily for 24 h without further stirring to form a greenish-spongy precipitate. The precipitate was filtered and washed several times with distilled water to remove settled salts, followed by washing with 0.1 M HCl to remove unreacted monomers. Finally, the precipitate was washed with acetone to remove the water from the nanocomposite and kept for drying under a dynamic vacuum for 48 h at 50 °C to achieve constant weight [30].

### 2.3. Characterization Techniques

The Fourier-transform infrared (FTIR) spectra of the polyaniline were recorded on a Perkin Elmer 1600 spectrophotometer in KBr medium in the wave number range 400–4600 cm^−1^. The prepared samples were ground with KBr in the ratio of 1:5 until it forms homogeneous powder, which is later pressed into a 10 mm plate with the help of a hydraulic press. The diffraction patterns were captured in the range 10–80° under 2 Theta using a Siemens D-5000 powder X-ray diffractometer with CuK_α_ source radiation at a wavelength of 1.54°. The surface morphology of polyaniline in the form of powder coated with gold particles by sputtering was investigated employing Philips XL 30 ESEM scanning electron microscope on a gold substrate. The contact angle is measured using the Theta Optical Tensiometers, NanoScience Instrument Ltd., Alexandria, VA, USA.

## 3. Results and Discussion

### 3.1. FTIR Spectra

**Figure 1:**
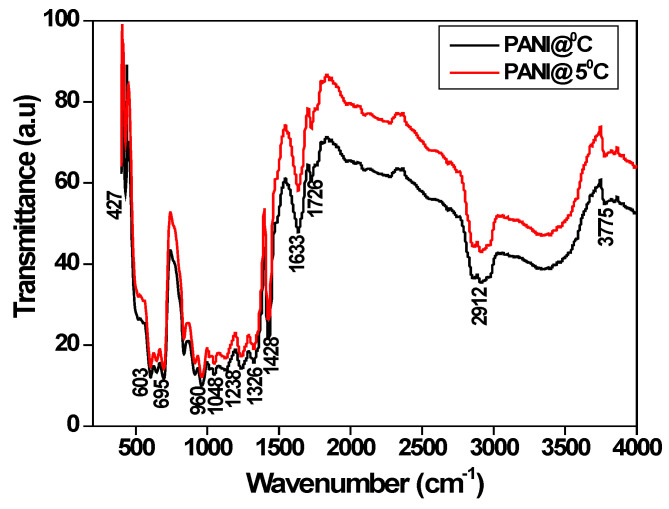
FTIR spectra of polyaniline prepared at different temperatures.

Figure 1 shows the spectra of PANI prepared at 5 °C and 0 °C temperatures by oxidation polymerization method. The characteristic absorption bands are seen at 427 cm^−1^, 603 cm^−1^, 695 cm^−1^, 960 cm^−1^, 1048 cm^−1^, 1238 cm^−1^, 1326 cm^−1^, 1428 cm^−1^, 1633 cm^−1^, 1726 cm^−1^, 2912 cm^−1^, and 3775 cm^−1^, respectively [31,32]. The broad band at 2912 cm^−1^ and 3775cm^−1^ is attributed to the O–H stretching of hydroxyl molecules. The bands that appeared at 1726 cm^−1^ correspond to N-H symmetry bending. Notably, 1633 cm^−1^ is due to the C=C stretching of the quinoid, and 1428 cm^−1^ is the characteristic and benzenoid rings. The bands at 1326 cm^−1^ and 1238 cm^−1^ are attributed to the C–N stretching of the aromatic benzenoid rings. The broadband at 1048 cm^−1^ for the “electronic–like band” is related to the vibration mode of N=Q=N (Q represents the quinoid ring), signifying the formation of HCl-doped PANI. The band at 960 cm^−1^ is associated with the out-of-plane deformation of C–H in the 1,4–disubstituted benzene ring, and 603 cm^−1^ and 695 cm^−1^ correspond to C–H bending out of the plane [33].

**Figure 2:**
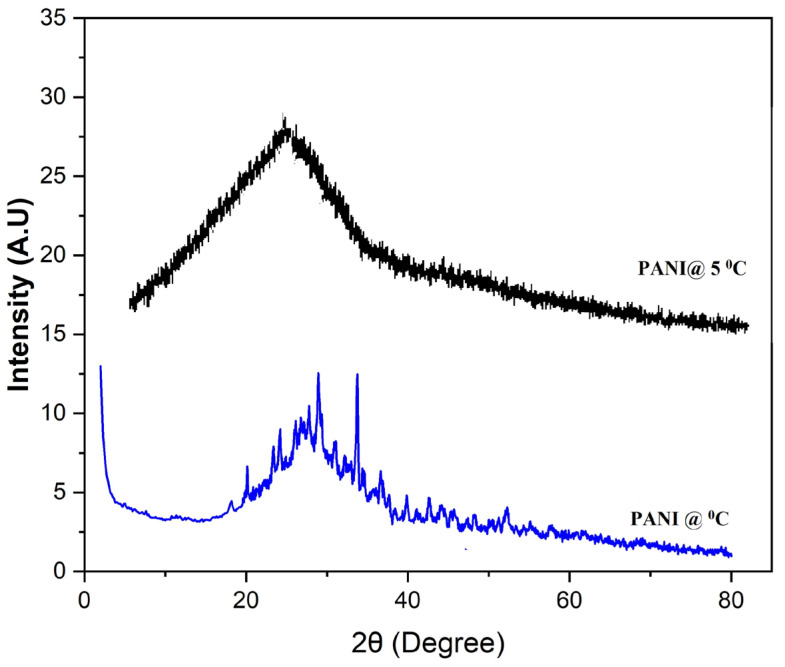
X-Rays diffraction pattern of polyaniline prepared at different temperatures.

Figure 2 shows the X-ray powder diffraction (XRD) patterns of PANI prepared in 0 °C and 5 °C. The 3 diffraction peaks of PANI at 2θ = 20.8°, 25.2°, and 33.69° correspond to (100), (110), and (111) planes due to the periodicity being parallel and perpendicular to the polymer chains. This suggests that PANI’s structure is semi-crystalline, similar to emerald salt prepared on ice. However, it is also noticed that polyaniline prepared at 5 °C is in the form of amorphous, and no peak appeared in the spectra. The obtained results with the ones from previously reported research work [34,35,36].

**Figure 3:**
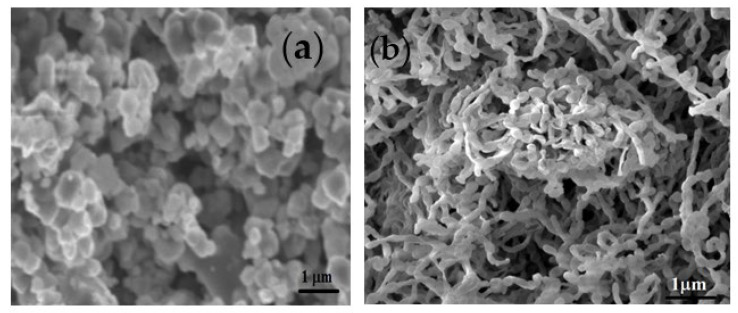
SEM Images of polyaniline prepared at 5 °C (**a**) and 0 °C (**b**) temperatures.

Figure 3 shows the SEM image of polyaniline prepared at 5 °C and 0 °C temperatures. It is observed from Figure 3a that the polyaniline prepared at 5 °C temperature without any surfactant forms granular shapes, well connected to each other. The polyaniline prepared in an ice bath produces natural fibers because the polymerization takes place inside the ice pores and the capillary action on polymer growth occurs in one dimension [37]. The polyaniline prepared in an ice medium form fiber in structure and its length is around 480 nm as seen in Figure 3b.

**Figure 4:**
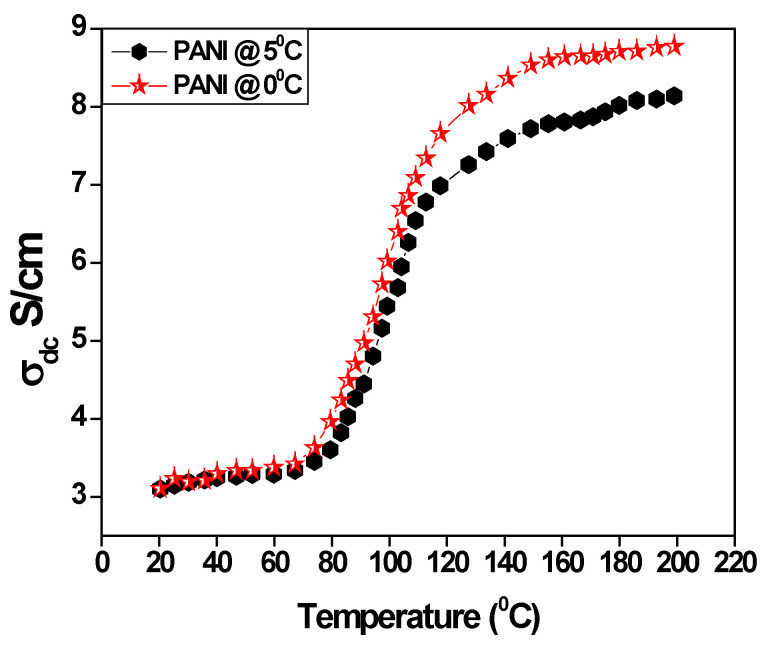
The DC conductivity of Polyaniline.

The effect of a change in morphology of the prepared polyaniline for temperature-dependent conductivity of polyaniline was studied in the temperature range 30°–220 °C, as shown in Figure 4. It is observed that the variation of conductivity is a function of temperature for polyaniline prepared at a varied temperature. The conductivity of polyaniline shows three steps increment in the graphs. In the initial stage, the conductivity is almost negligible variation in the temperature range of 30°–80 °C and then a rapid increase in conductivity from the temperature range of 80 °C to 140 °C. This behavior may be due to the thermal hopping of charge carriers between lower to higher conduction sites [38]. The exponential increment of conductivity is observed at the temperature range from 140 °C to 220 °C. This is significantly due to the extended polymer chain length and morphological changes of the polyaniline backbone where the coil-like structure changes into a linear structure. These conformational transform in the polymer backbone helps in the hopping of charge carriers, leading to enhanced conductivity [39]. It is important to note that the polyaniline prepared in the ice medium shows higher conductivity, i.e., 8.7 S/cm may be due to the fiber morphology of the polyaniline.

### 3.2. Dielectric Study

**Figure 5:**
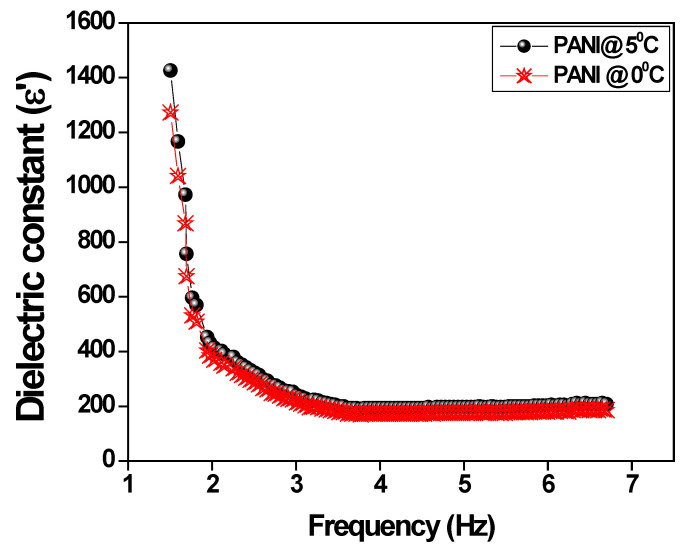
The variation of the dielectric constant versus the applied frequency.

The change of the dielectric constant as a function of applied frequency for polyaniline prepared at different temperatures is shown in Figure 5. It is observed that the dielectric constant decreases with an increase in frequency may be due to the blocking of charge carriers because of the interfacial effect within the polyaniline two layers and electrode effects. The dielectric constant is high at the lower frequency and gradually decreases with an increase in frequency and remains constant after 3 Hz. At high frequencies, the gradual reversal of the electric field takes place due to the fast ion diffusion in the same direction as the applied field; hence, it can no longer follow the field variation. It is also important to note that the dielectric constant decreased for the polyaniline prepared by surfactant Toluene-4-sulfonic acid monohydrate which helps the nulation of aniline monomers in an ice bath to form one-dimensional nanostructured polyaniline fiber, and, as a result, the dielectric constant is reduced compared to polyaniline prepared at optimal temperatures [40].

**Figure 6:**
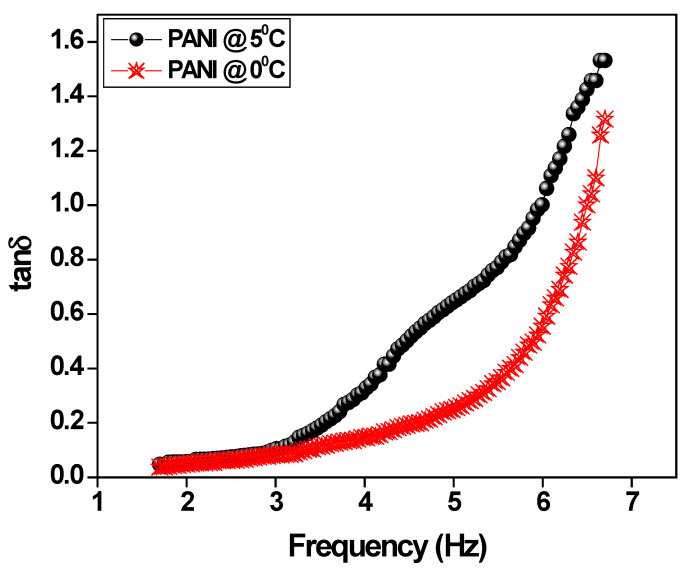
The variation of dielectric loss versus applied frequency for polyaniline.

The change of dielectric loss with respect to applied frequency for polyaniline is depicted in Figure 6. The dielectric losses are almost constant at a lower frequency of up to 3Hz then losses gradually increase with the increase of frequency due to the disturbance in the steady charge flow as a result of incident charges during the elliptical polarization occurring through the parallel polyaniline fibers. It is also important to notice that the polyaniline prepared without surfactant by a standard procedure forms a granular structure which is not favorable for the liner electronic polymerization and causes higher dielectric loss. Consequently, the polyaniline fiber prepared in an ice bath offers the easiest path for electron transportation, leading to a low loss in the polyaniline fiber [41,42,43].

**Figure 7:**
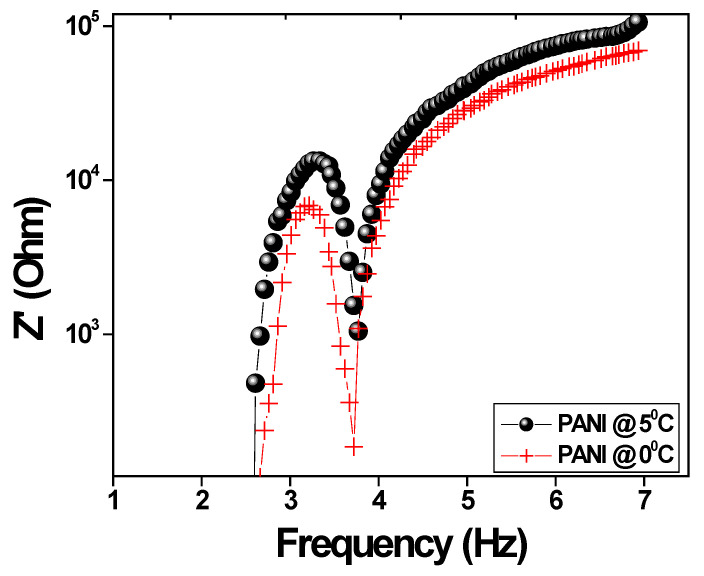
The variation of impedance versus applied frequencies.

The variation of impedance value with respect to applied frequencies for polyaniline is shown in Figure 7. The impedance spectra appeared in a semicircular shape, indicating the reduction of interfacial resistance and grain resistance of the polyaniline. As the area under the curve of the circle decreases, resistance also decreases because it forms series resistance with a capacitance circuit among the polymer grains [44]. The ohmic resistance in the phase angle has an asymptotic value below −90° at high frequency and is considered the ideal condition for polarization in the backbone of N–H in the polyaniline chain. Therefore, the fiber nature of polyaniline shows a lower impedance value and high conductivity.

**Figure 8:**
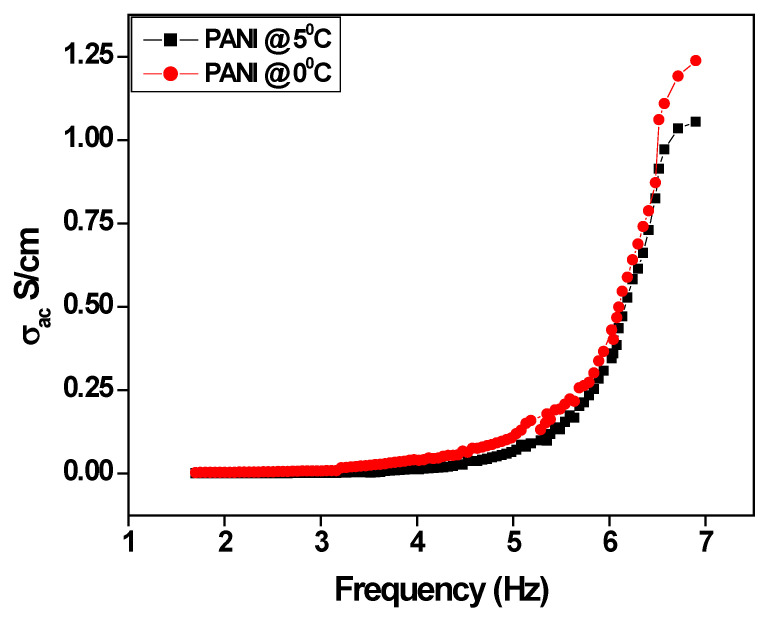
The variation of ac conductivity as a function of applied frequency.

Figure 8 shows the ac conductivity of polyaniline prepared at different temperatures. The conductivity increases with an increase in applied frequency due to the contribution of dielectric polarization at the lower frequency and electronic polarization results in slit dispersion at higher frequencies and reduces the grain resistance of the polyaniline fiber. The frequency-dependent conductivity shows three steps of conductivity at the initial stages. Conductivity is almost constant up to 3Hz beyond which it gradually increases, and, finally, after 4 Hz, conductivity jumps exponentially, which is also the characteristic peak of disordered semiconductor materials. It is observed that the polyaniline fiber has a higher conductivity of 1.23 S/cm compared to the bulk polyaniline [45,46,47].

### 3.3. Hydrogen Sorption Measurements of PANI Fibers

The volumetric hydrogen sorption measurements are important in understanding the hydrogen storage behavior of PANI fiber. Hydrogen absorption at 60 °C was performed at high pressure (H_2_ w 80 bar) with a pre-calibrated reservoir. The isothermal volumetric analyses were carried out by Hy-Energy’s PCTPro 2000 sorption equipment. This instrument is fully automated by Sievert’s uses, with an internal PID-controlled pressure regulator with a pressure of 170 bars. The hydrogen purging cycles, leak test, and PCT were studied by the HyDataV2.1 Lab-View program.

Padmapriya et al. [48] reported that surface morphology plays an important role in hydrogen storage. The hydrogen storage capacity was measured by an accurate quadrupole quartz crystal microbalance method at room temperature and 0.5 MPa pressure, and one-dimensional nanostructured polyaniline was found to exhibit a higher hydrogen storage capacity than the 2.66% compared with bulk granular polyaniline samples. The nanocomposite with a tubular structure also exhibits excellent reversibility of 0.019 wt%. It was also found that the total hydrogen storage capacity in the polyaniline-copper nanocomposite was 1.85 times that of pure copper. Singh et al. [49] studied the PANI-GO composite fabricated by in situ polymerization methods. It is observed that the storage of hydrogen is considerably compromised because the GO coated on the polyaniline fibers is reduced from 0.47 wt% to 1.94 wt% for the GO integrated into the polyaniline fibers.

**Figure 9:**
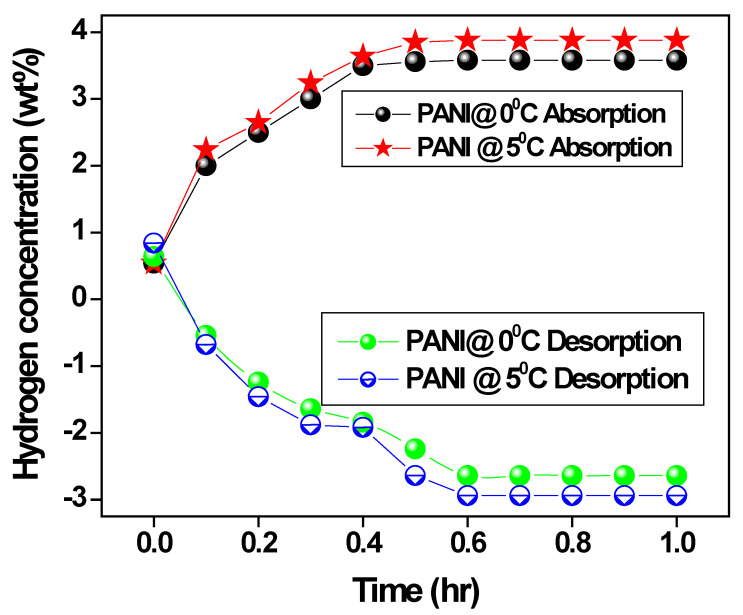
The absorption/desorption pattern of polyaniline.

Figure 9 shows the initial hydrogen uptake of polyaniline fiber prepared at optimum temperature and in an ice bath with respect to time. It is found that the absorption of polyaniline fiber was achieved, i.e., 86% of the total actual capacity of 8–8.5 wt% in less than 9 min by the polyaniline fiber. Further, the polyaniline fiber absorbed hydrogen was then desorbed when temperature increased at 60 °C against the pressure 1 bar approximately 1–2 wt% of hydrogen released at the optimum temperature. In order to confirm the efficiency of hydrogen sorption, the Pressure-Composition-Temperature (PCT) analysis was performed at room temperature for the polyaniline fiber. We found that a distinct variable pressure forms an effective hydride around 30 bar up to 2 wt% hydrogen uptake and a linear region of 2.5 wt% with a total absorption capacity of 3.5 wt% [50,51,52].

**Figure 10:**
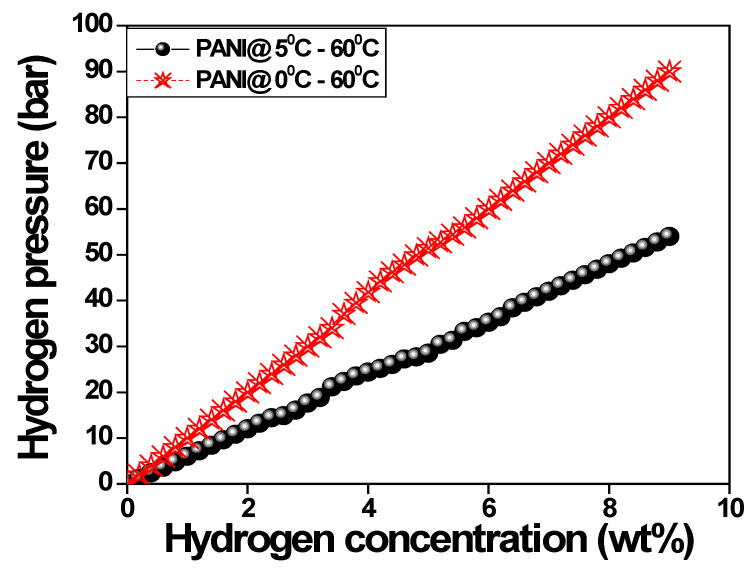
The variation of hydrogen absorption versus applied hydrogen pressure.

The anionic surfactant-based prepared polyaniline fiber was loaded into a Schlenk flask, sealed with a rubber cap, and dried in a vacuum at 50 °C for 1h. After the complete annealing of polyaniline samples were transferred to a high-pressure hydrogen reactor in a glove box filled with nitrogen gas. The completely sealed reactor was connected with a high hydrogen pressure volumetric setup to analyze the sorption performance. Figure 10 shows the variation of hydrogen absorption against applied pressure at 60 °C. It is observed that the hydrogen adsorption capacity increases with an increase in pressure. At room temperature, a hydrogen adsorption capacity is about 3.5 wt% whereas, at 60 °C, its capacity increases by 2-fold around 8.5 wt% for polyaniline fiber [53,54].

**Figure 11:**
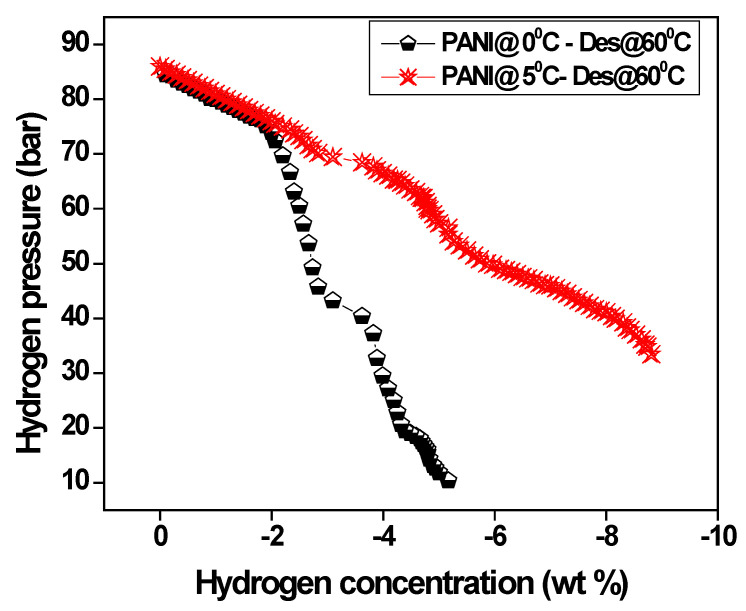
The variation of desorption of hydrogen gas versus applied pressure.

Figure 11 shows the variation of desorption of hydrogen gas against applied pressure. It is observed that hydrogen gas desorption occurs when pressure is slowly released, and lower temperature releases the hydrogen gases around 1.5 wt% when the pressure reduces further to 1 bar. The lower desorption of hydrogen gases from polyaniline fiber may be due to the strong affinity of protons with N = H bonding of the polymer backbone and the hygroscopic nature of the polyaniline.

## 4. Conclusions

Polyaniline fibers were prepared by the oxidation polymerization method in the presence of toluene-4-sulfonic acid as a surfactant in an ice medium. The prepared polyaniline fibers and bulk polyaniline are subjected to structural and surface morphology analysis. FTIR spectra indicate the formation of characteristic peaks at 1633 cm^−1^ for C=C stretching of the quinoid ring and 1428 cm^−1^ are the characteristic and benzenoid ring which confirms the formation of polyaniline. X-ray diffraction pattern indicates that the bulk polyaniline prepared at the optimum temperature is amorphous, whereas the polyaniline fibers prepared in an ice medium are semi-crystalline. The SEM images confirm that the polyaniline prepared in an ice medium form fiber in structure and its length is around 480 nm. DC conductivity study reveals that polyaniline fibers have high conductivity due to the hopping of polarons and extended polyaniline chain length. The frequency-dependent conductivity confirms that the liner electronic polarization at a higher frequency is responsible for higher conductivity. Hydrogen storage measurements confirm that the polyaniline fibers adsorbed around 86% of the total actual capacity of 8–8.5 wt% in less than 9 min, and desorption takes place at lower temperatures. The hydrogen gases are released around 1.5 wt% when the pressure reduces to 1 bar. Therefore, it follows that polyaniline fibers can be one of the potential candidates for hydrogen storage applications.

## Data Availability

Research data is available upon request.
